# Association of Ancillary Pathology Findings in Non-neoplastic Renal Parenchyma and Renal Outcomes of Robotic-Assisted Partial Nephrectomy

**DOI:** 10.3389/fsurg.2021.652524

**Published:** 2021-04-16

**Authors:** Laura E. Geldmaker, Amanda E. Kahn, Kevin A. Parikh, Ivan E. Porter, Daniela A. Haehn, Essa M. Bajalia, Qihui Zhai, Colleen T. Ball, David D. Thiel

**Affiliations:** ^1^Department of Urology, Mayo Clinic, Jacksonville, FL, United States; ^2^Department of Nephrology, Mayo Clinic, Jacksonville, FL, United States; ^3^Department of Laboratory Medicine and Pathology, Mayo Clinic, Jacksonville, FL, United States; ^4^Division of Biomedical Statistics and Informatics, Mayo Clinic, Jacksonville, FL, United States

**Keywords:** arteriosclerosis, fibrosis, glomerulosclerosis, histologic abnormality, robotic-assisted partial nephrectomy

## Abstract

**Background:** To evaluate robotic-assisted partial nephrectomy (RAPN) renal outcomes associated with ancillary pathology findings in non-neoplastic renal parenchymal tissue.

**Methods:** Tissue samples from 378 RAPNs were analyzed for glomerular disease (GD), vascular disease (VD), and tubulointerstitial disease (TD). One hundred and fifty-two patients were excluded due to insufficient non-neoplastic tissue for analysis and 4 patients were excluded due to calyceal diverticulum. Non-neoplastic tissue was evaluated for GD (negative, moderate, or global), VD (absent, mild, moderate, or severe), and TD (present or absent). Associations of ancillary pathology factors with patient characteristics were explored using the non-parametric Kendall tau-test and propensity score adjusted longitudinal mixed effects regression models were used to evaluate associations of these pathology factors with changes in estimated glomerular filtration rate (eGFR) following RAPN.

**Results:** One hundred and fifty-three (68.9%) patients had hypertension and 50 (22.5%) patients had diabetes. The majority of patients did not have any GD (*N* = 158, 71.2%) or TD (*N* = 186, 83.8%) while 129 (58.1%) had VD. VD was categorized as absent (*N* = 93, 41.9%), mild (*N* = 45, 20.3%), moderate (*N* = 76, 34.2%), and severe (*N* = 8, 6.8%). Older age (*P* = 0.018), hypertension (*P* < 0.001), and high grade MAP score (*P* = 0.047) were associated with a higher number of ancillary pathology factors. High grade MAP score (*P* = 0.03, *P* = 0.002) and hypertension (*P* = 0.02, *P* < 0.001) were individually associated with GD severity and VD severity, respectively. Older age was also individually associated with VD severity (*P* = 0.002) and hypertension was associated with TD (*P* = 0.04). Moderate-to-severe VD was associated with a worse change in eGFR from pre-RAPN to 1-month post-RAPN compared to those with mild or no VD (difference in mean change, −3.4 ml/kg/1.73m^2^; 95% CI, −6.6 to −0.2 ml/kg/1.73m^2^; *P* = 0.036).

**Conclusions:** Moderate-to-severe VD in non-neoplastic renal parenchyma is associated with post-operative changes in eGFR. Older age, hypertension, and high grade MAP scores are associated with the number of ancillary pathologies observed in RAPN specimens.

## Introduction

Nephron sparing surgery has become the favored treatment of small renal masses due to the preservation of non-neoplastic renal tissue which can delay or eradicate the onset of chronic kidney disease (CKD) in many patients (1). The prioritization of preserving healthy renal parenchyma has made partial nephrectomy, especially robotic-assisted partial nephrectomy (RAPN), more widely utilized for the treatment of renal masses.

**Figure 1:**
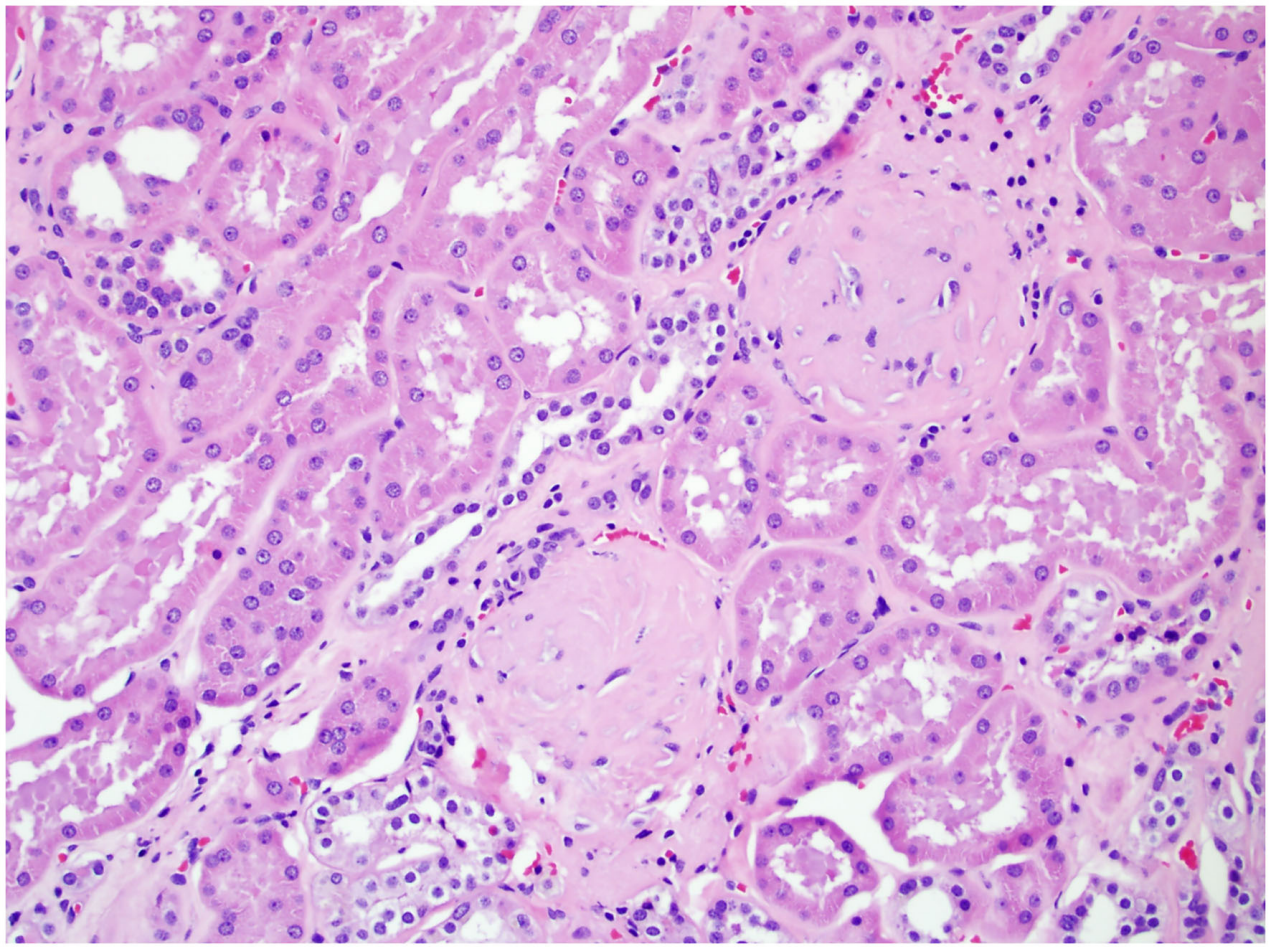
Non-neoplastic renal tissue from a patient with GD on 200× magnification. Two glomeruli were sclerotic, a typical histologic feature for glomeruli disease.

**Figure 2:**
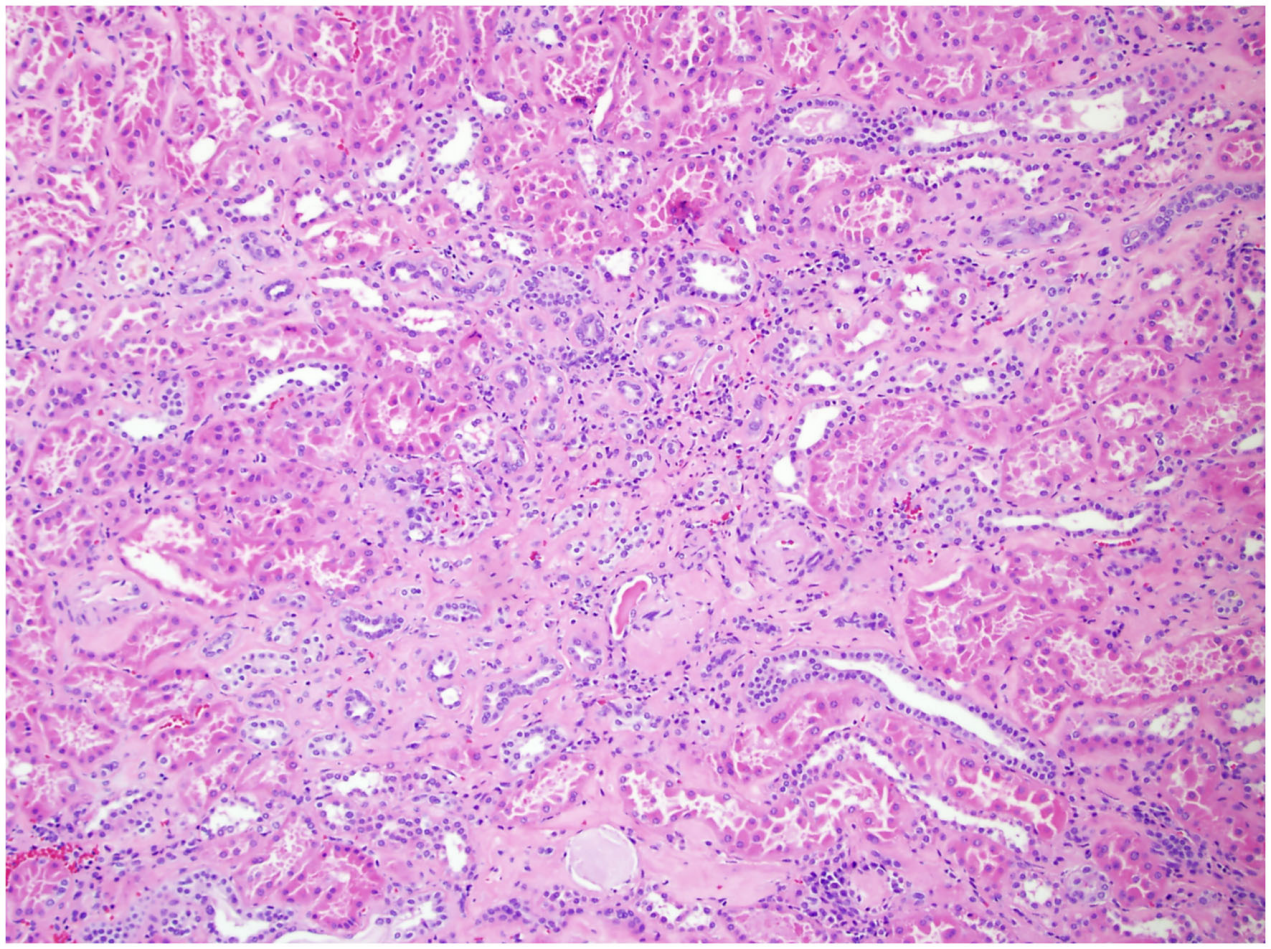
Non-neoplastic renal tissue on 100× of a patient with TD. Pictured here is interstitial disease, evidenced by the interstitial fibrosis and chronic inflammation.

**Figure 3:**
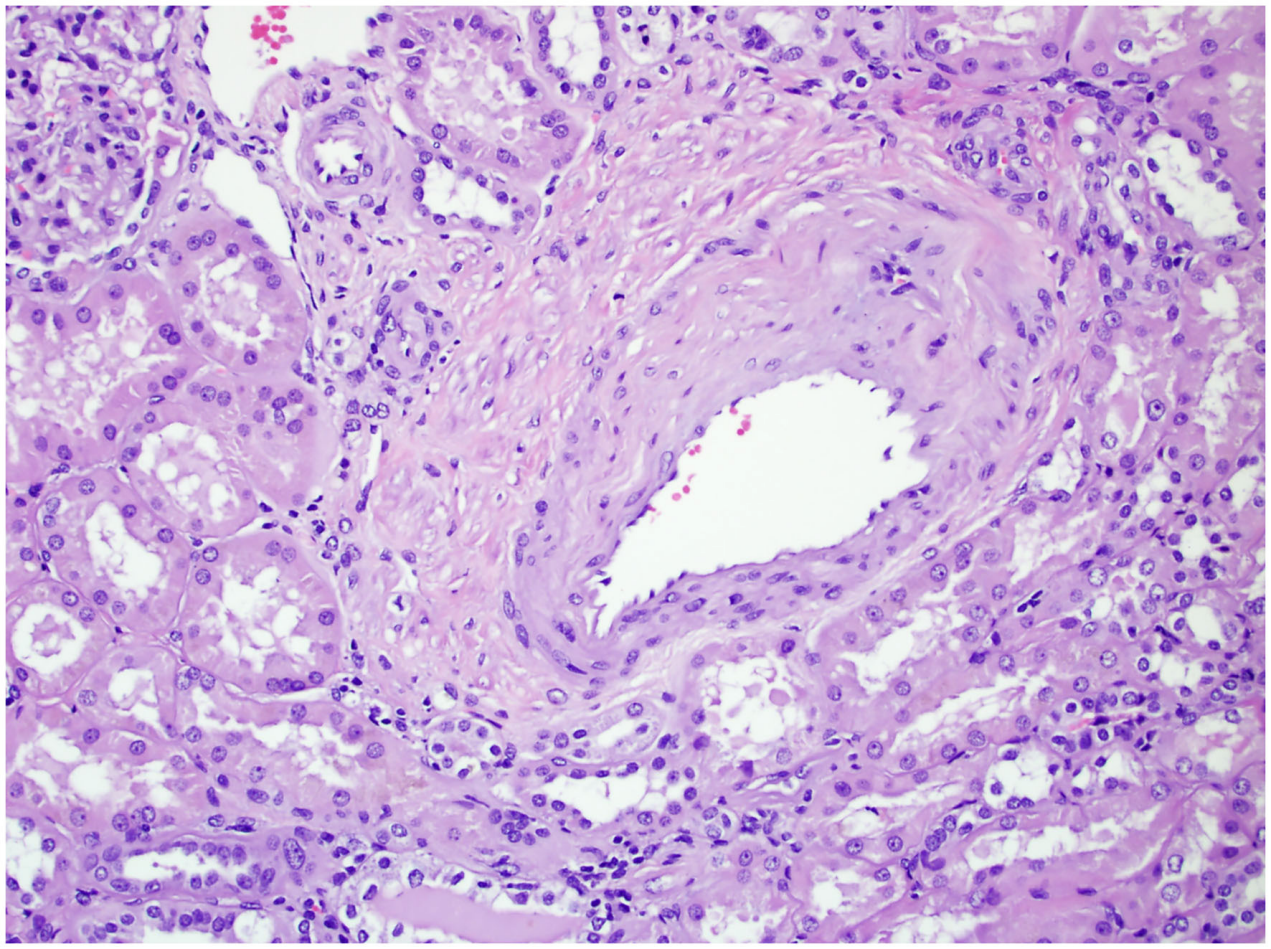
The non-neoplastic renal tissue of a patient with VD on 200× magnification. The figure shows renal arteriolar stenosis, evidenced by the narrowing of the vascular lumen and thickened vascular wall.

In 2010, the College of American Pathologists protocol mandated the evaluation of ancillary pathology in non-neoplastic renal parenchyma during radical and partial nephrectomy for glomerular disease (GD) (Figure 1), tubulointerstitial disease (TD) (Figure 2), and vascular disease (VD) (Figure 3) (2). It is unclear how the presence of these histologic abnormalities affects a patient's long-term renal function following RAPN.

Studies have evaluated the impacts of histological changes in non-neoplastic kidney tissue following radical nephrectomy (3–6). Additional studies have demonstrated a connection between diabetes mellitus (DM) and hypertension (HTN) with ancillary pathology observed in non-neoplastic renal tissue (7, 8). Motivated by these prior studies we examine the association between ancillary pathology findings in the non-neoplastic renal tissue (GD, VD, and TD) and post-operative estimated glomerular filtration rate (eGFR) in patients following RAPN. We also explore pre-operative variables that may be predictive of ancillary pathology in non-neoplastic renal parenchyma. We present the following article in accordance with the STROBE checklist.

## Methods

### Patient Selection and Tissue Analysis

Following institutional review board approval, 378 patients who underwent RAPN performed by a single, robotically trained surgeon from February 2008 through October 2018 were analyzed. One hundred and fifty-two patients were excluded due to insufficient non-neoplastic tissue for analysis and 4 patients were excluded due to calyceal diverticulum. Routine hematoxylin and eosin (H&E) stains were sufficient for most of the cases. Periodic acid-Schiff (PAS) and/or Jones methenamine silver stains were applied when necessary. A judgement of whether the amount of non-neoplastic renal parenchyma was sufficient for evaluation of medical kidney disease was made on a case by case basis. Five millimeters of non-neoplastic renal parenchyma for evaluation is a reasonable recommendation based on previous studies (9, 10). The pseudocapsule around the tumor may contain sclerotic glomeruli and tubular atrophy and may show fibrointimal thickening of arteries, followed by a zone of several millimeters of acute tubular injury, which may not be representative of the cortex elsewhere (11).

### Patient Analysis

Patient demographic data including patient's age, sex, weight, race, and body mass index (BMI) were collected. Additional pre-operative variables were collected including presence of HTN and DM, eGFR, R.E.N.A.L. nephrometry score (12), and Mayo Adhesive Probability (MAP) score (13). The CKD-EPI Creatinine 2009 equation was used to calculate eGFR (14). Data points following RAPN were collected to evaluate pathology results, non-neoplastic findings, and eGFR at 1 and 6 month time points.

Robotic-assisted partial nephrectomy was performed by one experienced surgeon using the robotic Da Vinci Si Surgical System as previously described (15, 16). Tumor size, tumor pathology, and non-neoplastic pathology were extracted from patient pathology reports generated by experienced genitourinary pathologists.

The non-neoplastic tissue was evaluated for histologic abnormalities and then categorized into three broad groups directed by the 2010 College of American Pathologists protocol: glomerular disease (GD), vascular disease (VD), and tubulointerstitial disease (TD). The GD category includes pathology of glomerulosclerosis, sclerotic or retracting glomeruli, diabetic glomerulomegaly, and other glomerular diseases of varying severities. In our study GD was described as negative (no glomeruli/nephrons affected), moderate (some mild to moderate or scattered GS or cortical scarring), or global (widespread or severe glomerular damage) to characterize severity. The VD category includes pathology identifying arteriosclerosis, arteriolosclerosis, arteriolonephrosclerosis, atherosclerosis, vascular hyalinosis, vasculopathy, and other vascular diseases of varying severity. VD was described as 0 (absent), 1 (mild), 2 (moderate), 3 (severe) for our study. The TD category includes pathology described as interstitial fibrosis, pyelonephritis, nephropathy, interstitial tubular atrophy, and other tubulointerstitial disease. TD was described as either present or absent. We chose to evaluate histologic abnormalities with broad categories to evaluate the clinical relevance of a review of ancillary pathology. Additionally, we analyzed the number of ancillary pathology factors a patient presented with (0, 1, 2, or 3) and the association with post-operative eGFR and comorbidities.

Post-operatively, patients were evaluated at 1 and 6 months for creatinine and eGFR measurements. Absolute change in eGFR (ACE) and percent change in eGFR (PCE) were calculated at all three time points. ACE and PCE were calculated with the following formulas: ACE = eGFR post-operative—eGFR pre-operative and PCE = [(eGFR post-operative—eGFR pre-operative)/(eGFR pre-operative)] ^*^ 100%. Compromised renal function was defined as a post-operative eGFR measurement that does not return within 10% of the pre-operative baseline eGFR measurement.

### Statistical Analysis

Numeric variables were summarized with the sample median and range. Categorical variables were summarized with the frequency and percentage of patients. Associations of ancillary pathology factors with patient characteristics were explored using the non-parametric Kendall tau test. Separately for each ancillary pathology factor (glomerular disease, tubulointerstitial disease, and moderate-to-severe vascular disease), we evaluated associations of the ancillary pathology factor with changes in eGFR from pre-operative to each post-operative time point (1 and 6 months) using a longitudinal mixed effects regression model with patient-specific random effects for intercepts and slopes. We included eGFR from pre-operative, 1 and 6 months in our model with indicator variables representing the two post-operative time points. Pre-operative eGFR (intercept) was modeled with the ancillary pathology factor. Changes in eGFR from baseline (slopes) were modeled with the ancillary pathology factor, warm ischemia time, and total operative time. Propensity score methods were used to improve balance in pre-operative characteristics of the patients in the two groups (patients with the ancillary pathology factor, patients without the ancillary pathology factor).

For each analysis comparing the change in eGFR of those with the ancillary pathology factor to those without the ancillary pathology factor, the propensity score (*P*_*i*_) was obtained for each patient (*i*). *P*_*i*_ was calculated as the expected probability of having the ancillary pathology factor from a multivariable logistic regression model using patient characteristics known prior to surgery (age, sex, race, BMI, hypertension, diabetes, R.E.N.A.L. score, and MAP score). Stabilized inverse probability weights (*w*_*i*_) were then calculated as $w_{i}=P^{*}/P_{i}$ for those with the ancillary pathology factor and $w_{i}=(1-P^{*})/(1-P_{i})$ for those without the ancillary pathology factor where P^*^ is the proportion of patients in our cohort with the ancillary pathology factor. Weighted standardized mean differences (SMD) were used to assess the level of balancing between groups where we considered SMDs > 0.10 to indicate imbalance between groups. All statistical tests were two-sided. Statistical analyses were performed using SAS (version 9.4; SAS Institute, Inc., Cary, North Carolina).

## Results

Characteristics of 222 patients with tissue available after RAPN are summarized in Table 1. R.E.N.A.L. scores and MAP scores are also reported in Table 1. The median pre-operative eGFR was 75 ml/min (range, 12–120 ml/min/1.73 m^2^) and 80.6% of patients (*N* = 179) had a pre-operative eGFR 60 ml/min/1.73 m^2^ or greater. Eighty-one patients (36.5%) did not have GD, VD, or TD noted in non-neoplastic renal tissue. A majority of patients did not have any GD (*N* = 158, 71.2%) or TD (*N* = 186, 83.8%). VD was described as absent in 41.9% of patients (*N* = 93), mild in 20.3% of patients (*N* = 45), moderate in 34.2% of patients (*N* = 76), and severe in 3.6% of patients (*N* = 8).

**Table 1:** Patient characteristics.

	**Summary (*N* = 222)**
**PRE-OPERATIVE CHARACTERISTICS:**	
Median age (range), years	63 (31–84)
**Age distribution, *n* (%)**	
31–64 years	122 (55.0%)
65–84 years	100 (45.0%)
**Sex, *n* (%)**	
Female	77 (34.7%)
Male	145 (65.3%)
Median body mass index (range), kg/m^2^	29.8 (19.0–55.1)
**Body mass index distribution, *n* (%)**	
19.0–24.9 kg/m^2^	34 (15.3%)
25.0–29.9 kg/m^2^	80 (36.0%)
30.0–55.1 kg/m^2^	108 (48.6%)
**Race, *n* (%)**	
White	188 (84.7%)
Black	25 (11.3%)
Asian	3 (1.4%)
Unknown	6 (2.7%)
**Hypertension, *n* (%)**	
No	69 (31.1%)
Yes	153 (68.9%)
**Diabetes, *n* (%)**	
No	172 (77.5%)
Yes	50 (22.5%)
**R.E.N.A.L. score, *n* (%)**	
4–6	51 (23.0%)
7–9	123 (55.4%)
10–12	48 (21.6%)
**MAP score, *n* (%)**	
0–3 Low grade	154 (69.4%)
4–5 High grade	68 (30.6%)
Median eGFR (range), mL/min/1.73 m^2^	75 (12–120)
**eGFR distribution, *n* (%)**	
60–144 mL/min/1.73 m^2^	179 (80.6%)
12–59 mL/min/1.73 m^2^	43 (19.4%)
**ANCILLARY PATHOLOGY:**	
**Glomerular disease (GD), *n* (%)**	
Negative	158 (71.2%)
Moderate	18 (8.1%)
Global	46 (20.7%)
**Tubulointerstitial disease (TD), *n* (%)**	
Absent	186 (83.8%)
Present	36 (16.2%)
**Vascular disease (VD), *n* (%)**	
Absent	93 (41.9%)
Mild	45 (20.3%)
Moderate	76 (34.2%)
Severe	8 (3.6%)
**Total number of pathology factors, *n* (%)**	
0	81 (36.5%)
1	67 (30.2%)
2	59 (26.6%)
3	15 (6.8%)

*MAP, Mayo Adhesive Probability; eGFR, estimated glomerular filtration rate. The sample median (minimum, maximum) is shown for numeric variables. Number (percentage of patients) is shown for categorical variables*.

Table 2 shows associations of the number of ancillary pathology factors per patient with pre-operative patient characteristics. Older age (*P* = 0.018), HTN (*P* < 0.001), and high grade MAP score (*P* = 0.047) were associated with a higher number of ancillary pathology factors. Each ancillary pathology factor (GD, VD, and TD) was individually evaluated for associations with the aforementioned patient characteristics. Increased severity of GD was associated with HTN (*P* = 0.023) and high grade MAP score (*P* = 0.027; Supplementary Table 1), presence of TD was associated with HTN (*P* = 0.042; Supplementary Table 2), and increased severity of VD was associated with older age (*P* = 0.002), HTN (*P* < 0.001), high grade MAP score (*P* = 0.022), and lower eGFR (*P* = 0.024; Supplementary Table 3). No other patient characteristics in Table 1 had a statistically significant association with GD severity (all *P* ≥ 0.10), TD (all *P* ≥ 0.082), or VD severity (all *P* ≥ 0.21).

**Table 2:** Associations of patient characteristics with the number of ancillary pathology factors.

	**Number of ancillary pathology factors**				
**Characteristic**	**0 Factors (*N* = 81)**	**1 Factors (*N* = 67)**	**2 Factors (*N* = 59)**	**3 Factors (*N* = 15)**	**Kendall Tau *P*-value**
Age, No. (%)					0.018
31–64 y	53 (65%)	31 (46%)	33 (56%)	5 (33%)	
65–84 y	28 (35%)	36 (54%)	26 (44%)	10 (67%)	
Sex, No. (%)					0.81
Female	28 (35%)	25 (37%)	19 (32%)	5 (33%)	
Male	53 (65%)	42 (63%)	40 (68%)	10 (67%)	
BMI, No. (%)					0.21
19.0–24.9 kg/m^2^	15 (19%)	9 (13%)	7 (12%)	3 (20%)	
25.0–29.9 kg/m^2^	30 (37%)	29 (43%)	16 (27%)	5 (33%)	
30.0–55.1 kg/m^2^	36 (44%)	29 (43%)	36 (61%)	7 (47%)	
Race, No. (%)					0.90
Black	9 (11%)	8 (12%)	7 (12%)	1 (7%)	
Non-black	72 (89%)	59 (88%)	52 (88%)	14 (93%)	
Hypertension, No. (%)					<0.001
No	38 (47%)	18 (27%)	10 (17%)	3 (20%)	
Yes	43 (53%)	49 (73%)	49 (83%)	12 (80%)	
Diabetes, No. (%)					0.92
No	62 (77%)	52 (78%)	48 (81%)	10 (67%)	
Yes	19 (23%)	15 (22%)	11 (19%)	5 (33%)	
R.E.N.A.L., No. (%)					0.81
4–6	20 (25%)	13 (19%)	13 (22%)	5 (33%)	
7–9	44 (54%)	40 (60%)	34 (58%)	5 (33%)	
10–12	17 (21%)	14 (21%)	12 (20%)	5 (33%)	
MAP score, No. (%)					0.047
Low grade	61 (75%)	49 (73%)	34 (58%)	10 (67%)	
High grade	20 (25%)	18 (27%)	25 (42%)	5 (33%)	
eGFR, No. (%)					0.35
12–59 mL/min	13 (16%)	13 (19%)	15 (25%)	2 (13%)	
60–144 mL/min	68 (84%)	54 (81%)	44 (75%)	13 (87%)	
Pathology, No. (%)					0.18
Benign	8 (10%)	9 (13%)	8 (14%)	4 (27%)	
Malignant	73 (90%)	58 (87%)	51 (86%)	11 (73%)	

*For continuous variable (age, BMI, and eGFR), P-values were based on the correlations with the continuous variable; however, we report the number and percentage of patients of pre-specified clinical categories for ease of interpretation. Race was categorized as black or non-black; the non-black race category included white (n = 188), Asian (n = 3), and unknown (n = 6)*.

A descriptive summary of baseline eGFR and changes in eGFR from baseline to 1 and 6 months post-operatively according to ancillary pathology factors are shown in Table 3. There was evidence suggesting the possibility that an increase in severity of VD is associated with a decrease in the PCE pre-operatively to 1 month post-operatively; the median PCE pre-operatively to 1 month post-operatively was −12.0% when VD was absent (*n* = 93), −10.9% with mild VD (*n* = 45), −15.9% with moderate VD (*n* = 76), and −19.8% with severe VD (*n* = 8). Our primary analysis consisted of evaluating associations of ancillary pathology factors with absolute changes in eGFR from baseline to 1 and 6 months after RAPN using longitudinal mixed effects regression models with stabilized inverse probability weights (Table 4). Moderate-to-severe VD was associated with a change in eGFR from baseline to 1 month post-RAPN that was 3.4 ml/kg/1.73 m^2^ lower than patients with mild or no VD (95% CI, 0.2–6.6 ml/kg/1.73 m^2^; *P* = 0.036). There was no evidence of an association of vascular disease with change in eGFR from pre-RAPN to 6-months post-RAPN (*P* = 0.99). We did not find any association of post-RAPN change in eGFR with GD (all *P* ≥ 0.67) or TD (all *P* ≥ 0.44). Supplementary Table 4 shows how the pre-operative characteristics in each analysis were balanced between groups before and after propensity score weighting (all weighted SMDs ≤ 0.10). The mean weights were near 1.00 for all three analyses.

**Table 3:** Ancillary pathology factors and kidney function before and after robotic assisted partial nephrectomy.

		**Baseline**	**1 month post-operative**			**6 months post-operative**		
**Ancillary pathology factor**	***N***	**eGFR**	**Absolute change in eGFR from baseline**	**Percent change in eGFR from baseline**	**Compromised**	**Absolute change in eGFR from baseline**	**Percent change in eGFR from baseline**	**Compro mised**
Glomerular disease		Median (range)	Median (range)	Median (range)	Fraction (%)	Median (range)	Median (range)	Fraction (%)
Negative	158	76.0 (12.1–119.7)	−9.4 (−39.1–20.5)	−12.0 (−45.0–28.5)	84/146 (57.5%)	−9.4 (−39.1–20.2)	−12.0 (−45.0–28.5)	70/119 (58.8%)
Moderate	18	72.6 (16.0–111.0)	−11.1 (−66.6–0.5)	−17.8 (−99.1–3.3)	11/16 (68.8%)	−13.4 (−27.3–0.0)	−18.3 (−32.0–0.0)	11/13 (84.6%)
Global	46	73.1 (44.3–105.9)	−7.2 (−25.7–15.4)	−12.0 (−36.4–17.5)	21/50 (52.5%)	−9.1 (−28.8–0.0)	−10.9 (−36.4–0.0)	13/25 (52.0%)
**Tubulointerstitial disease**								
Absent	186	75.4 (12.1–119.7)	−9.5 (−66.6–20.5)	−12.0 (−99.1–28.5)	100/169 (59.2%)	−9.4 (−39.1–20.2)	−12.0 (−45.0–28.5)	80/135 (59.2%)
Present	36	72.0 (16.0–111.2)	−6.2 (−30.5–8.7)	−10.0 (−40.9–12.2)	16/33 (48.5%)	−10.0 (−25.5–7.1)	−16.0 (−31.3–11.1)	16/22 (72.7%)
**Vascular disease**								
Absent	93	75.4 (12.1–119.7)	−7.9 (−39.1–20.5)	−12.0 (−45.0–28.5)	47/87 (54.0%)	−8.6 (−39.1–14.1)	−11.4 (−45.0–17.5)	40/72 (55.6%)
Mild	45	82.6 (43.6–111.2)	−9.2 (−27.1–20.5)	−10.9 (−36.4–28.5)	23/40 (57.5%)	−10.2 (−37.3–12.4)	−13.3 (−42.1–28.5)	19/29 (65.5%)
Moderate	76	71.7 (16.0–111.5)	−9.2 (−66.6–15.0)	−15.9 (−99.1–22.4)	41/67 (61.2%)	−9.5 (−29.7–20.2)	−15.9 (−35.9–23.6)	33/53 (62.3%)
Severe	8	62.1 (39.9–92.9)	−12.0 (−20.0–4.5)	−19.8 (−27.7–9.4)	5/8 (62.5%)	−15.6 (−27.3–0.0)	−18.3 (−29.4–0.0)	2/3 (66.7%)
**Total number of pathology factors**								
0	81	76.6 (12.1–119.7)	−8.0 (−39.1–13.5)	−10.9 (−45.0–28.5)	39/75 (52.0%)	−8.6 (−39.1–14.1)	−10.9 (−45.0–17.5)	34/62 (54.8%)
1	67	74.6 (28.7–111.5)	−10.4 (−34.8–20.5)	−14.9 (−44.0–28.5)	42/63 (66.7%)	−9.5 (−37.3–20.2)	−13.3 (−42.1–28.5)	33/51 (64.7%)
2	59	77.0 (41.2–111.2)	−8.6 (−66.6–15.4)	−13.7 (−99.1–17.5)	30/50 (60.0%)	−9.7 (−28.8–7.1)	−14.9 (−36.4–11.1)	21/36 (58.3%)
3	15	71.6 (16.0–95.8)	−2.0 (−27.9–8.7)	−2.4 (−40.9–12.2)	5/14 (35.7%)	−10.0 (−21.3–0.0)	−19.0 (−31.3–0.0)	6/8 (75.0%)

*eGFR, estimated glomerular filtration rate (ml/kg/1.73 m^2^). Compromised was defined as having a percent change in eGFR that does not return to within 10% of the pre-operative baseline. eGFR was not available for 20 patients at 1 month postop or for 45 patients at 6 months postop*.

**Table 4:** Associations of ancillary pathology factors with change in estimated glomerular filtration rate (eGFR) after RAPN.

**Ancillary pathology factor**	**Difference in mean change in eGFR from preop to 1 month post-RAPN (95% CI), ml/kg/1.73 m^**2**^**	***P***	**Difference in mean change in eGFR from preop to 6 months post-RAPN (95% CI), ml/kg/1.73 m^**2**^**	***P***
Glomerular disease (moderate or global vs. negative)	0.4 (−3.0, 3.8)	0.82	−0.9 (−4.8, 3.1)	0.67
Tubulo interstitial disease (present vs. absent)	1.6 (−2.6, 5.9)	0.44	0.2 (−4.7, 5.0)	0.95
Vascular disease (moderate-to-severe vs. mild or absent)	−3.4 (−6.6, −0.2)	0.036	0.0 (−3.5, 3.6)	0.99

*The difference in mean change in eGFR at 1 and 6 months after RAPN were estimated from longitudinal mixed effects regression models with patient-specific random effects for intercepts (pre-RAPN eGFR) and slopes (change in eGFR post-RAPN) using stabilized inverse probability weighting. All models included indicator variables for each post-operative time point. Pre-RAPN eGFR and changes in eGFR were modeled with the given ancillary pathology factor. Post-operative changes in eGFR were additionally modeled with warm ischemia time and operative time*.

## Discussion

The evaluation of non-neoplastic renal tissue following partial or radical nephrectomy has become a standard practice at many institutions. However, the full potential of what can be learned from ancillary pathology has not yet been realized or executed clinically. The existing literature shows that the detection of VD or GD in renal tissue is associated with worse renal function and a decrease in eGFR following nephrectomy (3, 4, 17). However, radical nephrectomy itself is a possible confounding factor for a decrease in post-operative eGFR (1, 3, 4). To better understand the impact of histologic abnormalities found in non-neoplastic tissue, we conducted a study with patients who exclusively underwent RAPN and evaluated the association between a patient's ancillary pathology, post-operative eGFR, and comorbidities.

Brandina et al. examined 65 patients who underwent radical nephrectomy and had non-neoplastic renal parenchyma specimens available for analysis (3). Each specimen was obtained as far from the tumor as possible and was evaluated for glomerulosclerosis (GS), arteriosclerosis (AS), and interstitial fibrosis (IF) with H&E stains. After multivariate logistic regression, only GS (*P* = 0.039) proved to be an independent predictor factor for new-onset CKD by evaluating post-operative eGFR. Additionally, Gautam et al. evaluated 49 patients who underwent laparoscopic radical nephrectomy and evaluated non-neoplastic tissue for GS, AS, IF, as well as tubular atrophy (4). This group evaluated eGFR changes by calculating the PCE from baseline to the patient's last follow up. The extent of GS was significantly associated with post-operative PCE (*P* = 0.034) reflecting the rate of renal function decrease. AS and IF/tubular atrophy were not associated with changes in eGFR. Similar to our study, Capitanio et al. found a correlation with age and hypertension to renal parenchymal disease in their cohort of 171 patients who underwent radical nephrectomy (18). However, they utilized a renal parenchymal disease scoring system, while we individually evaluated each disease in relation to patient characteristics.

Few studies have been conducted with open and laparoscopic partial nephrectomy to evaluate non-neoplastic pathology (7, 9, 17). The literature suggests choosing non-neoplastic tissue furthest from the mass to evaluate for ancillary pathology. The availability of adequate tissue is understandably a concern when evaluating excised lesions from partial nephrectomy rather than having ample non-neoplastic tissue to evaluate from a radically excised kidney. One hundred and fifty-two of our 378 patients were excluded from analysis secondary to not having enough non-neoplastic parenchyma for evaluation. A study by Garcia-Roig et al. states that the open partial nephrectomy technique generally provides adequate non-neoplastic tissue to appropriately evaluate for ancillary pathology (9). In this study, they evaluated tissue blocks from 49 patients and determined 91.8% (*N* = 45) had adequate non-neoplastic tissue available for analysis. The use of the open technique may have allowed for more non-neoplastic tissue to be excised intraoperatively. Robotic-assisted partial nephrectomy may be a more precise nephron sparing technique; therefore, the availability of adequate non-neoplastic tissue may be more limited. The amount of parenchymal tissue is limited by enucleation RAPN techniques. Our cohort of patients underwent tumor excision rather than enucleation. While the benefits of limiting excised parenchymal mass (EPM) with enucleation technique have certainly been debated, we utilize an excision technique for two main reasons. The first is to ensure a safe surgical margin. Additionally, as published by Bajalia et al., while EPM has an impact on short term global renal function, it does not seem to have an impact on long term renal function if EPM remains reasonable (19).

Studies with partial nephrectomy specimens have reported that AS or VD is associated with a decline in renal function (*P* = 0.01) (17); however, these studies also report on associations between ancillary pathology and HTN, CAD, and DM (7, 8, 17). With a study of both radical and partial nephrectomies, Tewari et al. supported the evaluation of non-neoplastic renal parenchyma to determine the presence of various comorbidities such as DM and HTN which they believe may be the driving force behind a patient's worsening CKD (8). We support these findings with our large evaluation of non-neoplastic tissue following RAPN. To our knowledge, our study is the largest study conducted exclusively with non-neoplastic renal parenchyma obtained from partial nephrectomies and the first evaluation of patients following RAPN. The exclusive cohort of RAPNs is significant because the procedure controls for many factors that could be considered confounding when analyzing post-operative outcomes.

We found significant associations between the number of ancillary pathology factors detected and HTN (*P* < 0.001) and high grade MAP score (*P* = 0.047). We also found significant associations between HTN and VD (*P* < 0.001), GD (*P* = 0.02), and TD (*P* = 0.04) individually. Mayo Adhesive Probability score was significantly associated with GD (*P* = 0.03) and VD (*P* = 0.02). The MAP score was initially developed to predict the presence of adherent perinephric fat on pre-operative imaging (13). It is a subjectively graded score from 0 to 5 that evaluates the thickness and quality of the fat surrounding the kidney. We believe the association between VD and GD and the MAP score may be driven by the same patient specific risk factors such as older age, high BMI, history of smoking, CAD or DM (13, 20). Patients older than 65 who have HTN and a high grade MAP score are more likely to have all three ancillary pathology factors than their younger, non-hypertensive counterparts. We also found that moderate-to-severe VD was associated with a worse change in renal function from pre-RAPN to 1-month following RAPN compared to those with mild or no VD.

Determined by a review of the literature and the results from our study, we believe the pathologic analysis of non-neoplastic parenchyma is valuable as a tool to expose undetected medical conditions that present in the renal tissues such as HTN, CAD, and DM. The early detection of these conditions provides medical teams with a unique opportunity for intervention to treat these chronic diseases in the early stages of development. This analysis of ancillary pathology has demonstrated that older patients with high grade MAP scores and HTN also frequently present with VD, GD, and TD. This observation may be a useful clinical tool when deciding if a patient should undergo radical or partial nephrectomy. Bhindi et al. recently published a risk analysis to assist in decision making between radical and partial nephrectomy and demonstrated that at times, the risks of partial nephrectomy can outweigh the benefits (21). They determined that older age, low pre-operative eGFR, worse pre-operative proteinuria, and presence of diabetes, HTN, and a solitary kidney were associated with worse long-term eGFR. However, this study failed to consider a patient's MAP score and ancillary pathology which may significantly influence post-operative renal function. It may be beneficial to consider the results from our study of ancillary pathology, as well as the results from the Bhindi et al. risk analysis when deciding between radical and partial nephrectomy for a cohort of patients who have moderate risk. For example, consider a patient who has three of the six risk factors as described by Bhindi et al. This patient is a 70-year-old male who has diabetes, HTN, and a high grade MAP score. According to our analysis, it is likely this patient has two or three pathologic variables present that would potentially impact his renal function post-operatively and could benefit from the decision to perform a partial nephrectomy contrary to the traditional choice of radical nephrectomy.

This study was not without limitations due to its retrospective nature. The study was conducted at a tertiary institution and every RAPN was performed by a single surgeon which may limit the generalizability of the study's results. Further, the categorization of results from ancillary pathology analysis was retrospective and broad which may result in less than optimal appointments as GD, VD, or TD. Around 10% (*N* = 21) of patients were lost to follow up at 1 month and 24% (*N* = 54) of patients were lost to follow up at 6 months which may impact the analysis of long-term eGFR. However, the longitudinal mixed effects regression models used all available data points in the analysis. Finally, several statistical tests were performed for this study without adjustment for multiple testing and the possibility of a type II error should be considered when interpreting the results.

## Conclusions

The presence of vascular disease in non-neoplastic renal tissue following RAPN is associated with post-operative changes in eGFR. Pre-operative diagnosis of hypertension, older age, and elevated MAP score are significantly associated with the number of ancillary pathologic factors present in non-neoplastic tissue.

## Data Availability Statement

The raw data supporting the conclusions of this article will be made available by the authors, without undue reservation.

## Ethics Statement

The studies involving human participants were reviewed and approved by Institutional Review Board Mayo Clinic. Written informed consent for participation was not required for this study in accordance with the national legislation and the institutional requirements.

## Author Contributions

LG, AK, and DT: conception and design. DT: administrative support and provision of study materials or patients. All authors: collection and assembly of data, data analysis and interpretation, manuscript writing, and final approval of manuscript.

## Conflict of Interest

The authors declare that the research was conducted in the absence of any commercial or financial relationships that could be construed as a potential conflict of interest.

## References

[1] Ghandour RA Danzig MR McKiernan JM. Renal cell carcinoma: risks and benefits of nephron-sparing surgery for T1 tumors. Adv Chronic Kidney Dis. (2015) 22:258–65. 10.1053/j.ackd.2015.03.006 26088069

[2] Cancer Protocols Checklists College of American Pathologists. (2010). Available online at: http://www.cap.org/apps/docs/committees/cancer/cancer_protocols/2009/Kidney_09protocol.pdf (accessed December 28, 2020).

[3] Brandina R Moreira Leite KR Gregorio EP Fernandes KBP Srougi M. Histologic abnormalities in non-neoplastic renal parenchyma and the risk of chronic kidney disease following radical nephrectomy. Urology. (2017) 100:158–62. 10.1016/j.urology.2016.09.041 27725235

[4] Gautam G Lifshitz D Shikanov S Moore JM Eggener SE Shalhav AL. Histopathological predictors of renal function decrease after laparoscopic radical nephrectomy. J Urol. (2010) 184:1872–6. 10.1016/j.juro.2010.06.145 20850146

[5] Wang X Liu Q Kong W Huang J Chen Y Huang Y. Pathologic analysis of non-neoplastic parenchyma in renal cell carcinoma: a comprehensive observation in radical nephrectomy specimens. BMC Cancer. (2017) 17:900. 10.1186/s12885-017-3849-5 29282004 PMC5745993

[6] Trevisani F Di Marco F Capitanio U Dell'Antonio G Cinque A Larcher A. Renal histology across the stages of chronic kidney disease. J Nephrol. (2021). 10.1007/s40620-020-00905-y. [Epub ahead of print]. 33394348

[7] Gorin MA Garcia-Roig M Garcia-Buitrago M Parra-Herran C Jorda M Ciancio G. Atherosclerosis within the non-neoplastic margin of partial nephrectomy specimens: implications for medical management. World J Urol. (2013) 31:1531–4. 10.1007/s00345-012-0978-y 23187761

[8] Tewari R Bajaj R Bharadwaj. Medical renal disease in tumor nephrectomies: the silent killer. Saudi J Kidney Dis Transpl. (2018) 29:50–6. 10.4103/1319-2442.225211 29456207

[9] Garcia-Roig M Gorin MA Parra-Herran C Garcia-Buitrago M Kava BR Jorda M. Pathologic evaluation of non-neoplastic renal parenchyma in partial nephrectomy specimens. World J Urol. (2013) 31:835–9. 10.1007/s00345-011-0720-1 21691720

[10] Henriksen KJ Meehan SM Chang A. Nonneoplastic kidney diseases in adult tumor nephrectomy and nephroureterectomy specimens: common, harmful, yet underappreciated. Arch Pathol Lab Med. (2009) 133:1012–25. 10.1043/1543-2165-133.7.1012 19642728

[11] Bonsib SM Pei Y. The non-neoplastic kidney in tumor nephrectomy specimens: what can it show and what is important? Adv Anat Pathol. (2010) 17:235–50. 10.1097/PAP.0b013e3181e3c02d 20574169

[12] Kutikov A Uzzo RG. The R.E.N.A.L. nephrometry score: a comprehensive standardized system for quantitating renal tumor size, location and depth. J Urol. (2009) 182:844–53. 10.1016/j.juro.2009.05.035 19616235

[13] Davidiuk AJ Parker AS Thomas CS Leibovich BC Castle EP Heckman MG. Mayo adhesive probability score: an accurate image-based scoring system to predict adherent perinephric fat in partial nephrectomy. Eur Urol. (2014) 66:1165–71. 10.1016/j.eururo.2014.08.054 25192968

[14] Levey AS Stevens LA Schmid CH Zhang YL Castro AF III Feldman HI. A new equation to estimate glomerular filtration rate. Ann Intern Med. (2009) 150:604–12. 10.7326/0003-4819-150-9-200905050-00006 19414839 PMC2763564

[15] Taylor AS Lee B Rawal B Thiel DD. Impact of fellowship training on robotic-assisted laparoscopic partial nephrectomy: benchmarking perioperative safety and outcomes. J Robot Surg. (2015) 9:125–30. 10.1007/s11701-015-0498-z 26531112

[16] Bhayani SB Figenshau RS. The Washington University Renorrhaphy for robotic partial nephrectomy: a detailed description of the technique displayed at the 2008 World Robotic Urologic Symposium. J Robot Surg. (2008) 2:139–40. 10.1007/s11701-008-0096-4 27628249

[17] Lifshitz DA Shikanov SA Razmaria AA Eggener SE Liao C Chang A. Clinical and histologic predictors of renal function decline after laparoscopic partial nephrectomy. J Endourol. (2011) 25:1435–41. 10.1089/end.2010.0646 21797760

[18] Capitanio U Larcher A Fallara G Trevisani F Porrini E Di Marco F. Parenchymal biopsy in the management of patients with renal cancer. World J Urol. (2021). 10.1007/s00345-020-03572-7. [Epub ahead of print]. 33385247

[19] Bajalia EM Parikh KA Haehn DA Kahn AE Ball CT Thiel DD. Determinants and implications of excised parenchymal mass on robotic-assisted partial nephrectomy outcomes. Urology. (2020) 145:141–6. 10.1016/j.urology.2020.08.023 32958224

[20] Bijol V Batal I. Non-neoplastic pathology in tumor nephrectomy specimens. Surg Pathol Clin. (2014) 7:291–305. 10.1016/j.path.2014.04.001 26837441

[21] Bhindi B Lohse CM Schulte PJ Mason RJ Cheville JC Boorjian SA. Predicting renal function outcomes after partial and radical nephrectomy. Eur Urol. (2019) 75:766–72. 10.1016/j.eururo.2018.11.021 30477983

