# Supplementary material for: Association of Ancillary Pathology Findings in Non-neoplastic Renal Parenchyma and Renal Outcomes of Robotic-Assisted Partial Nephrectomy
Source: Front Surg. 2021 Apr 16;8:652524. doi: 10.3389/fsurg.2021.652524 (PMC8085594; doi:10.3389/fsurg.2021.652524)
Supplement: Supplementary file 1 [file Data_Sheet_1.docx]

Supplementary Material

# Supplementary Tables

Supplemental Table 1. Associations of patient and tumor characteristics with glomerular disease

|  | Glomerular disease |  |
| --- | --- | --- |

| Characteristic | Negative (N=158) | Middle (N=18) | Global (N=46) | Kendall Tau P value |
| --- | --- | --- | --- | --- |

| Age, No. (%) |  |  |  | 0.10 |
| --- | --- | --- | --- | --- |
| 31-64 y | 93 (59%) | 7 (39%) | 22 (48%) |  |
| 65-84 y | 65 (41%) | 11 (61%) | 24 (52%) |  |
| Sex, No. (%) |  |  |  | 0.43 |
| Female | 57 (36%) | 7 (39%) | 13 (28%) |  |
| Male | 101 (64%) | 11 (61%) | 33 (72%) |  |
| BMI, No. (%) |  |  |  | 0.43 |
| 19.0-24.9 kg/m^2^ | 25 (16%) | 3 (17%) | 6 (13%) |  |
| 25.0-29.9 kg/m^2^ | 61 (39%) | 6 (33%) | 13 (28%) |  |
| 30.0-55.1 kg/m^2^ | 72 (46%) | 9 (50%) | 27 (59%) |  |
| Race, No. (%) |  |  |  | 0.71 |

| Black | 18 (11%) | 4 (22%) | 3 (7%) |  |
| --- | --- | --- | --- | --- |

| Non-black | 140 (89%) | 14 (78%) | 43 (93%) |  |
| --- | --- | --- | --- | --- |
| Hypertension, No. (%) |  |  |  | 0.023 |
| No | 57 (36%) | 1 (6%) | 11 (24%) |  |
| Yes | 101 (64%) | 17 (94%) | 35 (76%) |  |
| Diabetes, No. (%) |  |  |  | 0.61 |
| No | 124 (78%) | 13 (72%) | 35 (76%) |  |
| Yes | 34 (22%) | 5 (28%) | 11 (24%) |  |
| R.E.N.A.L., No. (%) |  |  |  | 0.96 |
| 4-6 | 36 (23%) | 3 (17%) | 12 (26%) |  |
| 7-9 | 90 (57%) | 6 (33%) | 27 (59%) |  |
| 10-12 | 32 (20%) | 9 (50%) | 7 (15%) |  |
| MAP score, No. (%) |  |  |  | 0.027 |
| Low grade | 117 (74%) | 9 (50%) | 28 (61%) |  |
| High grade | 41 (26%) | 9 (50%) | 18 (39%) |  |
| eGFR, No. (%) |  |  |  | 0.33 |
| 12-59 mL/min/1.73m^2^ | 28 (18%) | 5 (28%) | 10 (22%) |  |
| 60-144 mL/min/1.73m^2^ | 141 (82%) | 13 (72%) | 36 (78%) |  |
| Pathology, No. (%) |  |  |  | 0.13 |
| Benign | 17 (11%) | 4 (22%) | 8 (17%) |  |
| Malignant | 141 (89%) | 14 (78%) | 38 (83%) |  |
| For continuous variable (age, BMI, and eGFR), P values were based on the correlations with the continuous variable, however we report the number and percentage of patients of pre-specified clinical categories for ease of interpretation. | | | | |

Supplemental Table 2. Associations of patient and tumor characteristics with tubulointerstitial disease

|  | Tubulointerstitial disease | |  |  |
| --- | --- | --- | --- | --- |
| Characteristic | Absent (N=186) | Present (N=36) | Kendall Tau P value |  |
| Age, No. (%) |  |  | 0.082 |  |
| 31-64 y | 105 (56%) | 17 (47%) |  |  |
| 65-84 y | 81 (44%) | 19 (53%) |  |  |
| Sex, No. (%) |  |  | 0.84 |  |
| Female | 64 (34%) | 13 (36%) |  |  |
| Male | 122 (66%) | 23 (64%) |  |  |
| BMI, No. (%) |  |  | 0.23 |  |
| 19.0-24.9 kg/m^2^ | 28 (15%) | 6 (17%) |  |  |
| 25.0-29.9 kg/m^2^ | 72 (39%) | 8 (22%) |  |  |
| 30.0-55.1 kg/m^2^ | 86 (46%) | 22 (61%) |  |  |
| Race, No. (%) |  |  | 0.98 |  |
| Black | 21 (11%) | 4 (11%) |  |  |
| Non-black | 165 (89%) | 32 (89%) |  |  |
| Hypertension, No. (%) |  |  | 0.042 |  |
| No | 63 (34%) | 6 (17%) |  |  |
| Yes | 123 (66%) | 30 (83%) |  |  |
| Diabetes, No. (%) |  |  | 0.091 |  |
| No | 148 (80%) | 24 (67%) |  |  |
| Yes | 38 (20%) | 12 (33%) |  |  |
| R.E.N.A.L., No. (%) |  |  | 0.68 |  |
| 4-6 | 41 (22%) | 10 (28%) |  |  |
| 7-9 | 105 (56%) | 18 (50%) |  |  |
| 10-12 | 40 (22%) | 8 (22%) |  |  |
| MAP score, No. (%) |  |  | 0.70 |  |
| Low grade | 130 (70%) | 24 (67%) |  |  |
| High grade | 56 (30%) | 12 (33%) |  |  |
| eGFR, No. (%) |  |  | 0.55 |  |
| 12-59 mL/min/1.73m^2^ | 37 (20%) | 6 (17%) |  |  |
| 60-144 mL/min/1.73m^2^ | 149 (80%) | 30 (83%) |  |  |
| Pathology, No. (%) |  |  | 0.87 |  |
| Benign | 24 (13%) | 5 (14%) |  |  |
| Malignant | 162 (87%) | 31 (86%) |  |  |
| For continuous variable (age, BMI, and eGFR), P values were based on the correlations with the continuous variable, however we report the number and percentage of patients of pre-specified clinical categories for ease of interpretation. | | | | |

Supplemental Table 3. Associations of patient and tumor characteristics with vascular disease

|  | Vascular Disease | | | |  |
| --- | --- | --- | --- | --- | --- |
| Characteristic | Absent (N=93) | Mild (N=45) | Moderate (N=76) | Severe (N=8) | Kendall Tau P value |
| Age, No. (%) |  |  |  |  | 0.002 |
| 31-64 y | 58 (62%) | 28 (62%) | 34 (45%) | 2 (25%) |  |
| 65-84 y | 35 (38%) | 17 (38%) | 42 (55%) | 6 (75%) |  |
| Sex, No. (%) |  |  |  |  | 0.53 |
| Female | 32 (34%) | 20 (44%) | 23 (30%) | 2 (25%) |  |
| Male | 61 (66%) | 25 (56%) | 53 (70%) | 6 (75%) |  |
| BMI, No. (%) |  |  |  |  | 0.56 |
| 19.0-24.9 kg/m^2^ | 17 (18%) | 6 (13%) | 10 (13%) | 1 (13%) |  |
| 25.0-29.9 kg/m^2^ | 33 (35%) | 17 (38%) | 27 (36%) | 3 (38%) |  |
| 30.0-55.1 kg/m^2^ | 43 (46%) | 22 (49%) | 39 (51%) | 4 (50%) |  |
| Race, No. (%) |  |  |  |  | 0.47 |
| Black | 11 (12%) | 2 (4%) | 9 (12%) | 3 (38%) |  |
| Non-black | 82 (88%) | 43 (96%) | 67 (88%) | 5 (63%) |  |
| Hypertension, No. (%) |  |  |  |  | <0.001 |
| No | 40 (43%) | 18 (40%) | 10 (13%) | 1 (13%) |  |
| Yes | 53 (57%) | 27 (60%) | 66 (87%) | 7 (88%) |  |
| Diabetes, No. (%) |  |  |  |  | 0.43 |
| No | 67 (72%) | 40 (89%) | 60 (79%) | 5 (63%) |  |
| Yes | 26 (28%) | 5 (11%) | 16 (21%) | 3 (38%) |  |
| R.E.N.A.L., No. (%) |  |  |  |  | 0.59 |
| 4-6 | 23 (25%) | 11 (24%) | 15 (20%) | 2 (25%) |  |
| 7-9 | 51 (55%) | 23 (51%) | 45 (59%) | 4 (50%) |  |
| 10-12 | 19 (20%) | 11 (24%) | 16 (21%) | 2 (25%) |  |
| MAP score, No. (%) |  |  |  |  | 0.022 |
| Low grade | 70 (75%) | 34 (76%) | 46 (61%) | 4 (50%) |  |
| High grade | 23 (25%) | 11 (24%) | 30 (39%) | 4 (50%) |  |
| eGFR, No. (%) |  |  |  |  | 0.024 |
| 12-59 mL/min/1.73m^2^ | 16 (17%) | 3 (7%) | 20 (26%) | 4 (50%) |  |
| 60-144 mL/min/1.73m^2^ | 77 (83%) | 42 (93%) | 56 (74%) | 4 (50%) |  |
| Pathology, No. (%) |  |  |  |  | 0.21 |
| Benign | 9 (10%) | 7 (16%) | 11 (14%) | 2 (25%) |  |
| Malignant | 84 (90%) | 38 (84%) | 65 (86%) | 6 (75%) |  |
| For continuous variable (age, BMI, and eGFR), P values were based on the correlations with the continuous variable, however we report the number and percentage of patients of pre-specified clinical categories for ease of interpretation. | | | | | |

Supplemental Table 4. Standardized mean differences before and after inverse probability weighting

|  | Glomerular Disease | |  | Tubulointerstitial Disease | |  | Vascular Disease | |
| --- | --- | --- | --- | --- | --- | --- | --- | --- |
|  | SMD | Weighted SMD |  | SMD | Weighted SMD |  | SMD | Weighted SMD |
| Age | 0.30 | 0.03 |  | 0.38 | 0.05 |  | 0.50 | 0.09 |
| Body mass index | 0.05 | 0.01 |  | 0.10 | 0.00 |  | 0.07 | 0.02 |
| RENAL score | 0.00 | 0.01 |  | 0.06 | 0.03 |  | 0.01 | 0.06 |
| MAP score | 0.25 | 0.07 |  | 0.21 | 0.03 |  | 0.40 | 0.01 |
| Male sex | 0.10 | 0.04 |  | 0.04 | 0.10 |  | 0.17 | 0.03 |
| Black race | 0.01 | 0.03 |  | 0.01 | 0.04 |  | 0.15 | 0.00 |
| Hypertension | 0.40 | 0.04 |  | 0.40 | 0.00 |  | 0.68 | 0.04 |
| Diabetes | 0.08 | 0.04 |  | 0.29 | 0.04 |  | 0.00 | 0.01 |
| SMD, standardized mean difference; MAP, Mayo adhesive probability. SMDs > 0.10 were considered to indicate imbalance. The mean (standard deviation) for the stabilized inverse probability weights was 1.00 (0.25) for the glomerular disease analysis, 1.00 (0.21) for the tubulointerstitial disease analysis, and 0.99 (0.43) for the vascular disease analysis. | | | | | | | | |
